# Structural and functional implications of p53 missense cancer mutations

**DOI:** 10.1186/1757-5036-2-5

**Published:** 2009-06-26

**Authors:** Yuhong Tan, Ray Luo

**Affiliations:** 1 Department of Molecular Biology and Biochemistry, University of California, Irvine, CA 92697-3900, USA

## Abstract

Most human cancers contain mutations in the transcription factor p53 and majority of these are missense and located in the DNA binding core domain. In this study, the stabilities of all core domain missense mutations are predicted and are used to infer their likely inactivation mechanisms. Overall, 47.0% non-PRO/GLY mutants are stable (ΔΔG < 1.0 kT) and 36.3% mutants are unstable (ΔΔG > 3.0 kT), 12.2% mutants are with 1.0 kT < ΔΔG < 3.0 kT. Only 4.5% mutants are with no conclusive predictions. Certain types of either stable or unstable mutations are found not to depend on their local structures. Y, I, C, V, F and W (W, R and F) are the most common residues before (after) mutation in unstable mutants. Q, N, K, D, A, S and T (I, T, L and V) are the most common residues before (after) mutation in stable mutants. The stability correlations with sequence, structure, and molecular contacts are also analyzed. No direct correlation between secondary structure and stability is apparent, but a strong correlation between solvent exposure and stability is noticeable. Our correlation analysis shows that loss of protein-protein contacts may be an alternative cause for p53 inactivation. Correlation with clinical data shows that loss of stability and loss of DNA contacts are the two main inactivation mechanisms. Finally, correlation with functional data shows that most mutations which retain functions are stable, and most mutations that gain functions are unstable, indicating destabilized and deformed p53 proteins are more likely to find new binding partners.

PACS codes: 87.14.E-

## 1. Introduction

p53 is a transcription factor involved in DNA repair, growth arrest and apoptosis [1-3]. It plays a critical role in cell responses to many cancer-causing events. Upon activation of certain oncogenes, p53 can stop cell cycle and induce apoptosis [1-3]. It has been reported that 50% of human cancers contain mutations in p53 [4], among which 95% are in the DNA-binding core (DBC) domain [4]. More interestingly 75% of DBC mutations occur as single missense mutations [4], i.e. most cancer mutants are full-length proteins. This finding holds the promise of a new therapy, which intends to restore apoptosis in cancer cells by activating the full-length p53 [5].

The crystal structure of Cho *et al *offers the first hints for the molecular mechanisms of cancer mutations in the DBC domain [6]. Their structure shows that residues most frequently mutated in cancer are at or near the protein-DNA interface [6]; the two most frequently mutated residues (R248 and R273) directly contact DNA [6]; the remaining four hot spot residues (R175, R249, R282 and G245) appear to play a critical role stabilizing the structure of the DNA binding interface [6]. Based on Cho *et al*'s structure, Bullock et al analyzed a wide range of missense mutants focusing on protein folding stability and DNA binding affinity [7,8]. They found that mutation sites causing loss of protein stability are mostly located in the beta-sandwich and Zinc-binding region, and mutation sites causing loss of DNA binding are at or near the DNA-binding interface [7,8]. Their analysis shows that a large fraction of cancer mutations may reduce stability of the DBC domain, resulting in loss of p53 functions at the body temperature. Thus, it is crucial to understand stability changes upon mutations in the DBC domain. To date only about 30 cancer mutants have been measured and analyzed [7-10], but about 1,300 different mutations have been reported for the DBC domain in cancer patients [4], among which about 1,000 mutations are missense.

In this work, we intend to analyze the stabilities and molecular contacts of all missense cancer mutations in the DBC domain to infer their likely molecular mechanisms. We have first validated the accuracy of three independent theoretical methods for protein stability prediction, PBSA [11], DFIRE [12], and FOLDX [13] with respect to measured relative stabilities for the DBC domain [7-10] before a comprehensive stability prediction is made for missense cancer mutants in the IARC database [4]. Based on the comprehensive stability prediction, we have analyzed the stability correlations with sequence, structure, and molecular contacts, and the correlations with clinical and functional data.

## 2. Results and Discussion

### 2.1. Overview of Relative Stabilities by Missense Mutations

All IARC missense cancer mutations in the DBC domain, codons 96 to 289, are subject to three independent analyses by PBSA [11], DFIRE [12], and FOLDX [13]. Among a total of 1,006 missense mutations, 642 can be analyzed quantitatively, 152 mutations result in structural clash in homology models (see Method) or loss of Zinc binding, and 212 mutations involves PRO (from or to) and GLY (from). The last two groups of mutants can only be analyzed qualitatively. All computed relative stabilities are given in Additional file 1. In our analysis, a mutation is predicted to be stable (or unstable) only when at least two of the three predictions are consistent with each other. Among the 642 mutations analyzed quantitatively, 373 mutations are stable, i.e. ΔΔG ≤ 1 kT – at least 98% folded at 37°C (1 kT ≈ 0.6 kcal/mol, k is Boltzmann constant, and T is thermodynamic temperature); 136 are unstable, i.e. ΔΔG > 3 kT – at most 85% folded at 37°C; 97 mutations are with relative stabilities between 1 kT and 3 kT, i.e. mutations in the grey zone; and 36 mutants have inconsistent predictions by the three methods. Overall, we find 373 (36.3%) out of 794 non-PRO/GLY mutants (47.0%) are stable, 288 mutants are unstable (including Zinc-binding and clash mutants, see Method), 97 mutants (12.2%) are in the grey zone, and 36 mutants (4.5%) are without consistent predictions. Following the works of Fersht and co-workers [9,10], highly stable mutants (ΔΔG ≤ -3 kT) are also identified in the hope to use these as suppressor mutations (Table 1). Nevertheless, a more logical approach in search of suppressor mutations would be to analyze the mutation space of the DBC domain systematically, which is actively under way in this group. In the following, only single missense mutations are analyzed (17 multiple mutations are excluded).

**Table 1:** Highly stable mutations (ΔΔG ≤ -3 kT) and their predicted relative stabilities by PBSA, DFIRE, and FOLDX, respectively.

MUTANTS	SEC.	ΔΔG (PBSA)	ΔΔG (DFIRE)	ΔΔG (FOLDX)	No. CASE.
A159V	S4	-2.27	-1.83	-1.49	41
A161V	S4	-3.51	-1.98	-1.14	12
N235I	S8	-2.85	-1.68	-2.04	5
N239Y	L3	-2.36	-2.41	-2.17	8
T256I	S9	-1.22	-1.96	-1.84	3
S269I	S10	-1.95	-1.99	-0.91	1

The unit of ΔΔG is in kcal/mol. Second column shows secondary structure elements. The last column gives the number of reported cases in the IARC database.

### 2.2. Missense Mutation Matrix

Fractions of unstable and stable mutations are shown in Table 2 and Table 3, respectively. In Table 2, the mutation matrix shows that 13 mutation patterns (V→W, V→E, I→S, I→T, I→F, I→N, F→C, Y→S, Y→C, Y→H, Y→D, Y→N, and R→W) are always unstable, indicating their loss of stability is less likely due to local structural environments. Finally, Y (87.5%), I (72%), C (70%), V (67%), F (62.5%) and W (60%) are the most common amino acids before mutation in unstable mutants. W (94%), R (58%) and F (56%) are the most common amino acids after mutation in unstable mutants.

**Table 2:** Fractions of unstable mutants (> 3 kT) from codons 96 to 289 before and after substitution.

	G	A	V	L	I	S	C	T	M	F	Y	W	H	K	R	D	E	N	Q	ALL
A	0/4		1/6			0/4		1/7		1/1						3/7				0.21

V	10/12	7/12		6/12	1/6				6/8	3/5		**3/3**				3/5	**9/9**			0.67

L			0/9		0/3	0/1			0/5	3/6			2/4		6/8				4/6	0.36

I			0/6	1/4		**6/6**		**6/6**	1/3	6/6	1/1					1/1		**6/6**		0.72

S	0/7	0/3		1/2	1/5		0/11	0/10		1/7	2/3			1/1	2/6			1/5		0.15

C	5/8	0/1				4/10		0/1		6/7	7/9	7/8	1/1		9/10			0/1		0.70

T		0/10			2/11	0/8			1/3					1/5	1/2			0/6		0.11

M			0/6	1/5	1/6			2/5						4/6	3/4					0.34

F	1/1		2/4	3/5	1/3	4/5	**4/4**				0/2									0.625

Y	1/1					**6/6**	**6/6**			0/5			**7/7**	1/1		**7/7**		**6/6**	1/1	0.875

W	1/1			0/1		1/1	0/1								1/1					0.60

H			0/1	2/5							2/7				4/6	1/6		1/4	2/6	0.34

K				0/1				0/3	0/3			1/1			1/5		1/4	0/4	1/3	0.17

R	3/15			0/12	1/3	2/13	0/9	2/4	0/2	1/1	1/2	**6/6**	3/10	1/4				1/2	3/8	0.26

D	0/7	0/3	1/8		0/1						2/8		2/7		1/1		0/7	0/8		0.12

E	2/11	0/9	0/10	0/2										5/11	1/1	1/10			1/8	0.16

N					1/8	0/9		0/7	0/1	1/1	3/6		2/6	0/6		1/8				0.15

Q				2/5									1/6	0/4	0/6		0/2			0.13

ALL	0.34	0.18	0.08	0.30	0.17	0.365	0.32	0.26	0.32	0.56	0.47	0.94	0.44	0.34	0.58	0.39	0.45	0.36	0.375	

First column shows residues before mutation and first row shows residues after mutation. Each element means fraction of unstable mutations. For example, there are a total of 3 mutations from I to M, only one of them is unstable. There are a total of 6 mutations from Y to S. All of them are unstable. Elements with 100% unstable mutations are highlighted in bold. The last column shows the percentage of total unstable mutations from a given WT amino acid. For example, mutations from WT V residue result in 67% unstable core domain. The last row shows the percentage of total unstable mutations to a given amino acid. For example, 94% mutations to W are unstable.

**Table 3:** Fractions of stable mutants (< 1 kT) from codons 96 to 289 before and after substitution.

	G	A	V	L	I	S	C	T	M	F	Y	W	H	K	R	D	E	N	Q	ALL
A	2/4		5/6			3/4		6/7		0/1						3/7				0.655

V	1/12	1/12		6/12	5/6				1/8	1/5		0/3				2/5	0/9			0.24

L			5/9		2/3	1/1			1/5	3/6			1/4		1/8				0/6	0.33

I			0/6	3/4		0/6		0/6	1/3	0/6	0/1					0/1		0/6		0.10

S	**7/7**	**3/3**		1/2	4/5		**11/11**	**10/10**		6/7	1/3			0/1	4/6			3/5		0.67

C	1/8	0/1				3/10		1/1		0/7	2/9	0/8	0/1		1/10			0/1		0.14

T		9/10			9/11	3/8			2/3					3/5	1/2			3/6		0.67

M			5/6	4/5	5/6			2/5						1/6	1/4					0.56

F	0/1		1/4	1/5	1/3	1/5	0/4				**2/2**									0.25

Y	0/1					0/6	0/6			**5/5**			0/7	0/1		0/7		0/6	0/1	0.125

W	0/1			0/1		0/1	0/1								0/1					0.0

H			1/1	3/5							5/7				2/6	2/6		2/4	3/6	0.51

K				1/1				**3/3**	2/3			0/1			4/5		2/4	3/4	2/3	0.71

R	3/15			11/12	1/3	4/13	5/9	2/4	1/2	0/1	1/2	0/6	5/10	3/4				0/2	3/8	0.43

D	3/7	**3/3**	6/8		1/1						5/8		4/7		0/1		6/7	7/8		0.70

E	4/11	6/9	8/10	1/2										4/11	0/1	6/10			4/8	0.53

N					7/8	8/9		**7/7**	1/1	0/1	2/6		3/6	4/6		6/8				0.73

Q				3/5									5/6	3/4	5/6		**2/2**			0.78

ALL	0.31	0.58	0.62	0.63	0.76	0.365	0.52	0.74	0.36	0.38	0.47	0.0	0.44	0.47	0.38	0.43	0.45	0.43	0.375	

First column shows residues before mutation and first row shows residues after mutation. Each element means fraction of stable mutations. Elements with 100% stable mutations are highlighted in bold. The last column shows the percentage of total stable mutations from a given WT amino acid. The last row shows the percentage of total stable mutations to a given amino acid. See Table 2 for more detail.

Table 3 shows the fractions of stable mutations. We found that 10 types of mutations (S→G, S→A, S→C, S→T, F→Y, Y→F, K→T, D→A, N→T, and Q→E) are always stable. These stable mutations do not depend on their environment. Finally, Q (78%), N (73%), K (71%), D (70%), S (67%), T (67%) and A (66%) are the most common amino acids before mutation in stable mutants. I (76%), T (72%), L (63%) and V (62%) are the most common amino acids after mutation in stable mutants.

### 2.3. Correlations between Sequence/Structure and Stabilities

**Figure 1:**
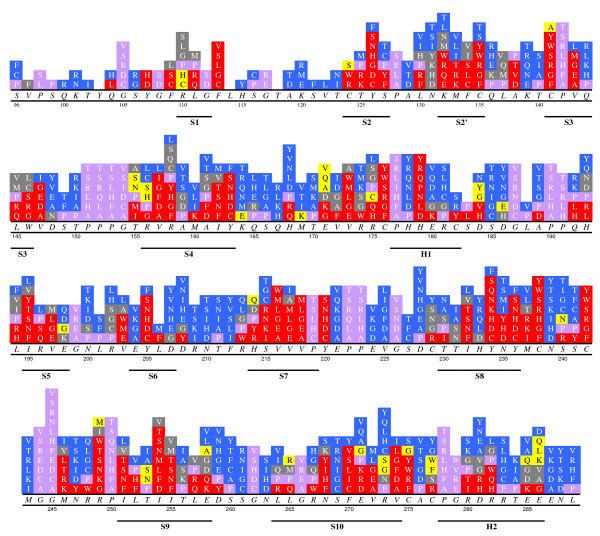
**Stability distribution of IARC missense mutations from codons 96 to 289**. Symbols in Italic Times: wild type amino acids; those in Normal Times: mutant amino acids; and those in Bold Times: secondary structures. Red grids: mutations with relative stabilities larger than 3 kT (including clash and Zinc-binding). Blue grids: mutations with relative stabilities less than 1 kT. Grey grids: mutations with relative stabilities between 1 kT and 3 kT. Mauve grids: mutations from or to PRO and from GLY. Yellow grids: mutations without consistent predictions.

**Figure 2:**
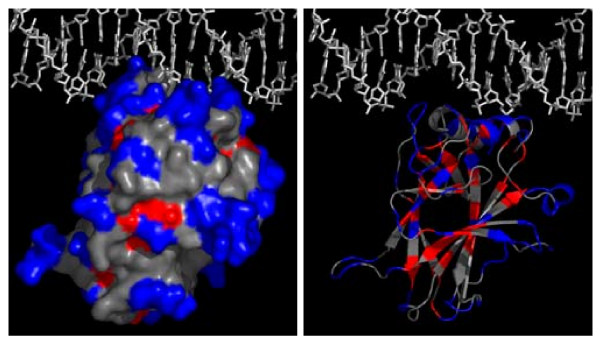
**Surface distribution of stable sites (in blue) and unstable sites (in red) (A, left). **Secondary structure distribution of stable sites (in blue) and unstable sites (in red) (B, right).

**Figure 3:**
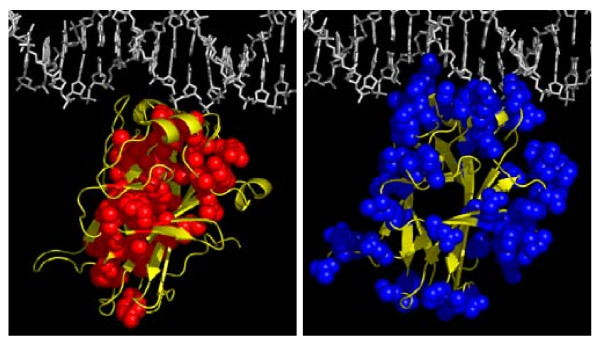
**Side chain exposures of unstable sites (A, left) and stable sites (B, right)**.

Stability distribution over sequence is shown in Fig. 1. It can be found that certain mutation sites are always stable or unstable no matter what amino acids are involved. Sites with more than 50% unstable mutations are defined as unstable below. Stable sites are also defined similarly. Fig. 2A shows surface distributions of stable and unstable sites, colored by blue and red, respectively. It is interesting to note that many stable sites are far from the DNA binding region. These mutations are less likely to disrupt DNA binding. Thus folding stability and binding affinity are probably not the only mechanisms for p53 inactivation by missense mutations. The likely causes of inactivation by these stable missense mutations will further be discussed below. Distributions of stable and unstable sites over secondary structures are shown in Fig. 2B. Visual inspection does not lead to noticeable correlation between secondary structures and stabilities. Finally Fig. 3 shows side chain exposures of unstable and stable sites, respectively. Not surprisingly almost all unstable sites are buried. This is consistent with the fact that most buried sites are hydrophobic so that mutations at these sites mostly disrupt hydrophobic packing. Indeed 93% unstable sites are buried by more than 80%, and 86% are buried by more than 90%. In contrast, most stable sites are exposed and 82% stable sites are exposed by more than 20%.

### 2.4. Correlation between Molecular Contacts and Stabilities

**Figure 4:**
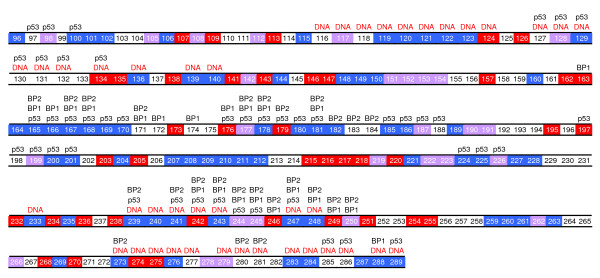
**Distribution of molecular contacts**. The DNA contacts (as in 2AC0, 2ADY, 2AHI, 2ATA (16)) are denoted by 'DNA', the tetramer subunit contacts (as in 2AC0, 2ADY, 2AHI, 2ATA (16)) are denoted by 'p53', the contacts with p53-binding protein 1 (as in 1GZH (17) and 1KZY (18)) or 2 (as in 1YCS (19)) are denoted by 'BP1' or 'BP2', respectively. Unstable sites are shown by red grids; stable sites by blue grids; Pro/Gly residues on wild type by mauve grids.

As shown above many stable cancer mutations are far from the DNA binding region. One possible reason that these DBC mutations inactivate p53 may be loss of protein-protein contacts with other p53 domains, with its tetramer subunits, or with its binding partners. It has been pointed out that specific p53-DNA binding is highly cooperative and involves interaction of p53 not only with the DNA, but also with other tetramer subunits [14]. Thus protein-protein interactions are critical in the formation of a stable p53-DNA complex, and have been implicated in DNA binding and the architectural accommodation of four DBC domains to a single recognition element [14,15]. In addition, the tetramer DBC domain is stabilized both by interactions within each dimer and by interactions between dimers [16]. Such interactions are critical in stabilizing functional p53-DNA complexes in cases where specific DNA interactions are diminished as a result of truncated DBC domains [16]. Fig. 4 shows molecular contact (DNA contacts also listed) distributions of the p53 DBC domain based on crystal structures [16-19]. Here two residues are defined to be in contact when any atom in one residue is within 6 Å of any atom in the other residue. Many molecules are know to bind p53 but no complex structures are available [20-25]. This limits our molecular contact analysis. Thus only contacts observed in available structures are analyzed and shown in Fig. 4. Nevertheless, 57.3% stable sites are for protein or DNA contacts (25% for protein-only contacts, 19.1% for DNA-only contacts, 13.2% for both protein and DNA contacts). These data imply that loss of protein contact may be a reason for p53 to lose its function upon stable mutations far from the DNA binding region.

All PRO/GLY sites are highly conserved in all mammalian p53 DBC domains so that their roles in structural integrity of the DBC domain can be assumed to be highly important. Existing DBC domain structures [6,16] are consistent with this assumption. Indeed measurements by the Fersht group show that P151S is 4.49 kcal/mol and G245S is 1.21 kcal/mol less stable than the WT DBC domain [7]. From Fig. 4, it can be found that many PRO and GLY sites, especially GLY sites (53.8%), are also involved in molecular contacts.

We found that most protein-protein contact sites (44.1%) are stable (Fig. 4). Consistent with previous analysis from the Fersht group [7,8], most DNA contact mutations (53.7%) are also stable (Fig. 1 &4). However, a few mutations are both in contact with DNA and unstable. Combining Fig. 1 and Fig. 4, it can be concluded that p53 inactivation may be due to: 1) loss of stability; 2) loss of DNA contact, protein-protein contact, or Zinc contact; 3) loss of stability and loss of DNA/protein-protein contact. Note that the above classification still cannot cover all missense cancer mutants. This results from our limited accuracy in stability prediction and limited structural data in molecular contact analysis. Nevertheless, it is clear that to activate p53 missense cancer mutants, we need to restore not only stability but also molecular contacts, especially protein-protein contacts.

### 2.5. Correlations between Cancer Types/Functions and Stabilities

**Figure 5:**
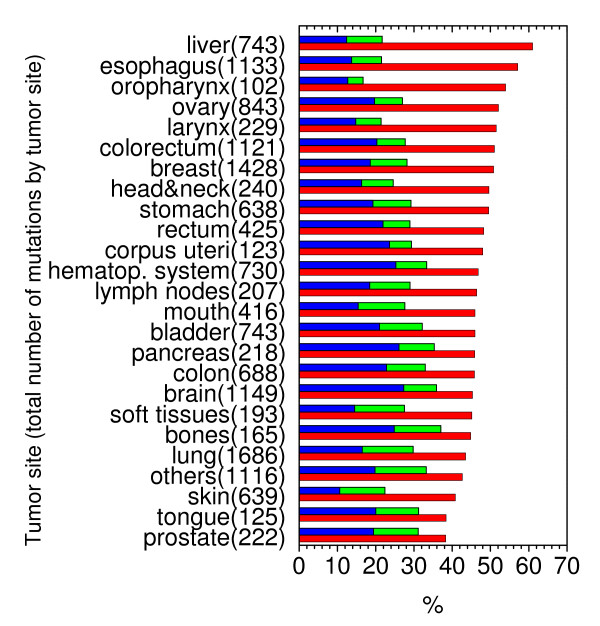
**Percentages of unstable (red), stable and DNA contact (blue), and stable and no DNA contact (green) mutations at different tumor sites**. Total number of mutations is also given by each tumor site.

**Figure 6:**
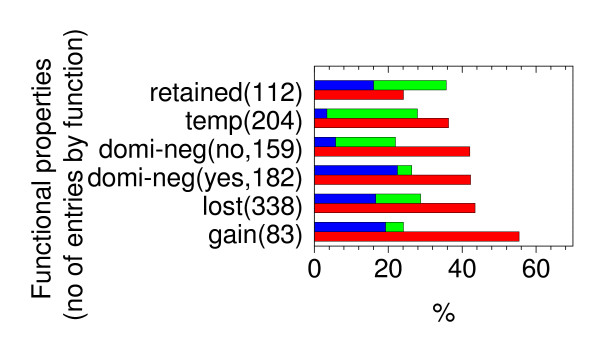
**Percentages of unstable (red), stable and DNA contact (blue), and stable and no DNA contact (green) mutations in different functional properties**. Retained: functional property of the mutant is similar to that of the WT; temp: the activity of the mutant is affected by the temperature at which the experiment is performed; domi-neg (no): dominant negative activity that the mutant does not counteract the effects of the WT under the control of a p53-responsive element when the two proteins are co-expressed in human or yeast cells; domi-neg (yes): the mutant protein counteract (or has a partial inhibiting effect on) the activity of the WT; lost: the WT functional property is lost by the mutant; gain: functional properties displayed by the mutant is not displayed by the WT protein. The total number of mutations is given by each functional property.

Finally we present the distributions of unstable or stable mutations in different cancers in Fig. 5. It can be found that most reported clinical cases contain unstable mutations. Since many stable mutations are in contact with DNA (see Fig. 4) (32.3%), loss of protein folding stability and DNA binding affinity are the main mechanisms in cancers involving missense mutations, consistent with the previous experimental annotation efforts by the Fersht group. Fig. 6 shows the percentages of unstable or stable mutations in functional analysis of missense mutations. Not surprisingly, most mutations that retain functions are stable (35.7%). Interestingly most mutations that gain functions are unstable (55.4%), indicating destabilized and deformed p53 proteins are more likely to find new binding partners. It is worth to point out that the interplay between stability and function is quite general in biomolecular recognition as analyzed in a recent theoretical study [26].

### 2.6. Limitations

Finally, it is necessary to discuss the limitations of the pure thermodynamics analysis that was utilized here for efficiency. Indeed, the effect of mutations on folding kinetics cannot be captured with thermodynamics analysis alone. Recent studies by Mahanty et al. [27,28] suggest mutations may interfere with folding kinetics even if they can increase stability. Thus whether stabilized mutations eventually lead to functionally more robust protein cannot be understood with the thermodynamics analysis alone [29-31].

## 3. Methods

Protein stability change upon mutation is defined as the folding free energy difference between a mutant protein (MT) and the wild type protein (WT):



Here, the folding free energy of a protein is the free energy difference between its native state (N) and denatured state (D): Δ *G*'_N-D _= *G*'_N_-*G*'_D_, with ' = MT or WT Prediction of protein stabilities has attracted much attention in computational biology with many computational models proposed [12,32-35] that take into account various factors important for protein stability [36-41].

In this work, we have chosen three well-established computational methods to predict protein stability change upon mutations. The first method is PBSA [11,42-44] that can be used to estimate stability if we assume hydrophobic and electrostatic (salt-bridge) interactions are the predominant components in stabilizing proteins. Here the hydrophobic free energy is estimated by a term linearly proportionally to the solvent accessible surface area (SA), and the electrostatic free energy is computed by solving the Poisson-Boltzmann (PB) equation [45]. Similar to protein pKa computations, the protein dielectric constant is set to be 20 to address electronic polarization, rotational polarization, and ionization effect. [46-48] The solvent dielectric constant is 80. The solvent ionic strength is set to be 150 mM. All calculations were performed at temperature 283 K. The second method is DFIRE by Zhou and Zhou [12] who have constructed a new residue-specific all-atom potential of mean force from nonhomologous protein structures with their proposed new reference state. The third method is FOLDX by Guerois *et al *[13] who have developed an efficient all-atom free energy function with weighted free energy terms trained using empirical data from experimental stabilities. After a control study of three models with available experimental data for the DBC domain, we use all three methods to comprehensively analyze the missense cancer mutants in the IARC database.

Crystal structure (codons 96 to 289), b chain of 1TSR [6] is used for the native state of the p53 DBC domain. Homology models are obtained by SCWRL3 [49] for the native state of missense mutants. If necessary, the denatured state is modeled as a tripeptide centered at the mutation site.

In this work, mutations causing structural clash and disruption of Zinc binding-sites (176, 179, 238, and 242) are assumed to be unstable by more than 3 kT. Indeed, these mutants are generally more unstable as observed in experiment (with ΔΔG ranging from 2.75 to 4.78 kcal/mol for F134L, V157F, H168R, R175H, M237I, I255F, R282W, T123A, H168R, and C242S [7,9,50]. Mutations from PRO/GLY and mutations to PRO are not covered in quantitative analysis because such mutations generally cause significant backbone entropy changes that cannot be modeled without time-consuming molecular dynamics analysis. However, they are analyzed qualitative based on sequence comparison of all mammalian p53.

**Figure 7:**
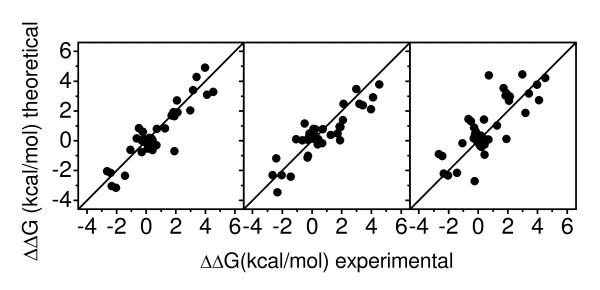
**Correlations between the three theoretical calculations and the experimental measurements by the Fersht group**. From left to right, the figure is for PBSA, DFIRE and FOLDX, respectively.

Quantitative agreements between experimental data and theoretical data from PBSA, DFIRE, and FOLDX, respectively, are shown in Fig. 7. Corresponding linear correlation coefficients are 0.91, 0.89 and 0.79, respectively; root mean square deviations are 0.80, 0.88, 1.25 kcal/mol, respectively; and unsigned average errors are 0.62, 0.75, 0.98 kcal/mol, respectively. In this work, three qualitative predictions are used to analyze all missense mutants: 1) relative stabilities less than 1 kT; 2) relative stabilities larger than 3 kT; and 3) relative stabilities less than -3 kT – highly stable mutations. Success rates of these qualitative predictions are also given in Table 4. The overall accuracies for the 1 kT prediction are 93%, 85%, and 85% by PBSA, DFIRE, and FOLDX, respectively; those for the 3 kT prediction are 96%, 91%, 93%, respectively; and those for the -3 kT prediction are 98%, 96%, 91%, respectively. Overall, excellent accuracies can be achieved by the three theoretical methods.

**Table 4:** Accuracies for qualitative predictions, by PBSA, DFIRE, and FOLDX, respectively.

		PBSA	DFIRE	FOLDX
≤ 1 kT	TP	91%	83%	89%
	
	TN	96%	87%	81%
	
	ACCU.	93%	85%	85%

> 3 kT	TP	100%	100%	90%
	
	TN	93%	87%	96%
	
	ACCU.	96%	91%	93%

≤ -3 kT	TP	80%	75%	50%
	
	TN	100%	98%	95%
	
	ACCU.	98%	96%	91%

TP: accuracy for true positive predictions; TN: accuracy for true negative predictions; ACCU: accuracy for overall accuracy combining TP and TN.

## 4. Conclusion

Most human cancers contain mutations in the transcription factor p53 and majority of these are missense and located in the DNA binding core domain. In this study, the stabilities of all core domain missense mutations are predicted and are used to infer their likely inactivation mechanisms. Overall, 47.0% non-PRO/GLY mutants are stable (ΔΔG < 1.0 kT) and 36.3% mutants are unstable (ΔΔG > 3.0 kT), 12.2% mutants are with 1.0 kT < ΔΔG < 3.0 kT. Only 4.5% mutants are with no conclusive predictions. Certain types of either stable or unstable mutations are found not to depend on their local structures. Y, I, C, V, F and W (W, R and F) are the most common residues before (after) mutation in unstable mutants. Q, N, K, D, A, S and T (I, T, L and V) are the most common residues before (after) mutation in stable mutants. The stability correlations with sequence, structure, and molecular contacts are also analyzed. No direct correlation between secondary structure and stability is apparent, but a strong correlation between solvent exposure and stability is noticeable. Our correlation analysis shows that loss of protein-protein contacts may be an alternative cause for p53 inactivation. Correlation with clinical data shows that loss of stability and loss of DNA contacts are the two main inactivation mechanisms. Finally, correlation with functional data shows that most mutations which retain functions are stable, and most mutations that gain functions are unstable, indicating destabilized and deformed p53 proteins are more likely to find new binding partners.

## Supplementary Material

Additional file 1 Quantitative stability predictions of all missense mutants. Click here for file

## References

[B1] Prives C, Hall P (1999). Journal of Pathology.

[B2] Vogelstein B, Lane D, Levine A (2000). Nature.

[B3] Vousden K (2000). Cell.

[B4] Olivier M, Eeles R, Hollstein M, Khan M, Harris C, Hainaut P (2002). Human Mutation.

[B5] Hupp T, Lane D, Ball K (2000). Biochemical Journal.

[B6] Cho Y, Gorina S, Jeffrey PD, Pavletich NP (1994). The Protein Data Bank Science.

[B7] Bullock A, Henckel J, Fersht A (2000). Oncogene.

[B8] Bullock AN, Fersht AR (2001). Nat Rev Cancer.

[B9] Nikolova P, Wong K, DeDecker B, Henckel J, Fersht A (2000). EMBO Journal.

[B10] Nikolova P, Henckel J, Lane D, Fersht A (1998). Proceedings of the National Academy of Sciences of the United States of America.

[B11] Tan YH, Luo R (2008). Journal Of Physical Chemistry B.

[B12] Zhou H, Zhou Y (2002). Proteins – Structure, Function and Genetics.

[B13] Guerois R, Nielsen J, Serrano L (2002). Journal of Molecular Biology.

[B14] Weinberg R, Veprintsev D, Fersht A (2004). Journal of Molecular Biology.

[B15] Nagaich A, Zhurkin V, Sakamoto H, Gorin A, Clore G, Gronenborn A, Appella E, Harrington R (1997). Journal of Biological Chemistry.

[B16] Kitayner M, Rozenberg H, Kessler N, Rabinovich D, Shaulov L, Haran TE, Shakked Z (2006). Mol Cell.

[B17] Derbyshire D, Basu B, Serpell L, Joo W, Date T, Iwabuchi K, Doherty A (2002). EMBO Journal.

[B18] Joo W, Jeffrey P, Cantor S, Finnin M, Livingston D, Pavletich N (2002). Genes & Development.

[B19] Gorina S, Pavletich N (1996). Science.

[B20] Weinberg R, Veprintsev D, Bycroft M, Fersht A (2005). Journal of Molecular Biology.

[B21] Buchhop S, Gibson M, Wang X, Wagner P, Sturzbecher H, Harris C (1997). Nucleic Acids Research.

[B22] Friedler A, Veprintsev D, Rutherford T, von Glos K, Fersht A (2005). Journal of Biological Chemistry.

[B23] Leng R, Lin Y, Ma W, Wu H, Lemmers B, Chung S, Parant J, Lozano G, Hakem R, Benchimol S (2003). Cell.

[B24] Uramoto H, Izumi H, Nagatani G, Ohmori H, Nagasue N, Ise T, Yoshida T, Yasumoto K, Kohno K (2003). Biochemical Journal.

[B25] Hansson L, Friedler A, Freund S, Rudiger S, Fersht A (2002). Proceedings of the National Academy of Sciences of the United States of America.

[B26] Wang J, Xu L, Wang E (2007). Biophys J.

[B27] Mohanty S, Hansmann UHE (2008). Journal of Physical Chemistry B.

[B28] Mohanty S, Meinke JH, Zimmermann O, Hansmann UHE (2008). Proceedings of the National Academy of Sciences of the United States of America.

[B29] Lu Q, Lu HP, Wang J (2007). Physical Review Letters.

[B30] Lu Q, Wang J (2008). Journal of the American Chemical Society.

[B31] Wang J, Lu Q, Lu HP (2006). Plos Computational Biology.

[B32] Topham C, Srinivasan N, Blundell T (1997). Protein Engineering.

[B33] Bordner A, Abagyan R (2004). Proteins – Structure, Function and Bioinformatics.

[B34] Bash P, Singh U, Langridge R, Kollmann P (1987). Science.

[B35] Tidor B, Karplus M (1991). Biochemistry.

[B36] Pace C, Shirley B, McNutt M, Gajiwala K (1996). FASEB Journal.

[B37] Dill K (1990). Biochemistry.

[B38] Ponnuswamy P (1993). Progress in Biophysics & Molecular Biology.

[B39] Rose G, Wolfenden R (1993). Annual Review of Biophysics and Biomolecular Structure.

[B40] Fersht A, Serrano L (1993). Current Opinion in Structural Biology.

[B41] Matthews B (1993). Annual Review of Biochemistry.

[B42] Honig B, Nicholls A (1995). Science.

[B43] Luo R, David L, Gilson MK (2002). Journal of Computational Chemistry.

[B44] Lu Q, Luo R (2003). Journal of Chemical Physics.

[B45] Gilson M, Honig B (1988). Proteins – Structure, Function and Genetics.

[B46] Schutz CN, Warshel A (2001). Proteins-Structure Function and Genetics.

[B47] Gilson MK (1995). Current Opinion in Structural Biology.

[B48] Honig B, Sharp K, Yang AS (1993). Journal of Physical Chemistry.

[B49] Canutescu A, Shelenkov A, Dunbrack R (2003). Protein Science.

[B50] Bullock A, Henckel J, DeDecker B, Johnson C, Nikolova P, Proctor M, Lane D, Fersht A (1997). Proceedings of the National Academy of Sciences of the United States of America.

